# A Retrospective Chart Review of Chronic Wound Patients Treated with Topical Oxygen Therapy

**DOI:** 10.1089/wound.2017.0729

**Published:** 2017-05-01

**Authors:** Karen Copeland, Angie R. Purvis

**Affiliations:** ^1^ Boulder Statistics, LLC, Boulder, Colorado.; ^2^ Angie Purvis, LLC, Evergreen, Colorado.

**Keywords:** chronic wounds, diabetes, wound management, wound healing, amputation, topical oxygen therapy

## Abstract

**Objective:** Topical oxygen devices are Food and Drug Administration (FDA) cleared for the following indications for use of various etiologies: skin ulcerations due to diabetes, venous stasis, postsurgical infections and gangrenous lesions, decubitus ulcers; amputations/infected stumps; skin grafts; burns; and frostbite. The goal of this study was to understand the impact of topical oxygen therapy (TOT) on patient outcomes, including amputation and healing rates.

**Approach:** This retrospective chart review included records collected between January 1, 2007, and July 18, 2016, from male and female patients ranging in age from 4 years to 105 years. All wounds were at least 1 cm^2^ and were treated with at least one separate modality before treatment with TOT and then treated with TOT for a minimum of 2 weeks in compliance with the FDA-approved indications. All records were from wounds that were no longer being treated with TOT.

**Results:** In this study, TOT was associated with an overall rate of 59.4% for a reduction in chronic wound size, while 41.6% of wounds had no healing. The overall amputation rate was 2.4% for wounds in this study.

**Innovation:** To our knowledge, this retrospective chart review represents one of the largest data sets (4,127 total wounds) collected over one of the longest time periods (9.5 years) to evaluate patient outcomes following TOT.

**Conclusion:** This study revealed healing and amputation rates similar to those reported in controlled clinical studies using TOT to treat chronic wounds.

**Figure:**
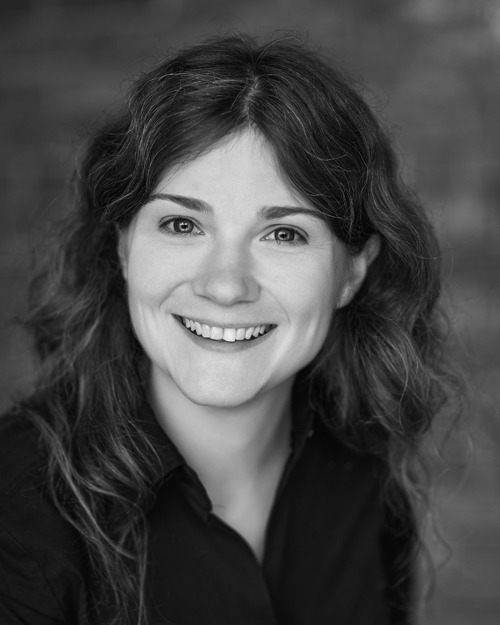
**Angie R. Purvis, PhD**

## Introduction

Chronic wounds are a rising healthcare issue in the United States and worldwide as the population ages and experiences increases in the incidence of diabetes and obesity. Approximately $50 billion (U.S.) are spent on chronic wound treatment annually^1,2^ and there are additional unknown social costs from lower quality of life, loss of productivity, pain and suffering, and burdens to caregiving family members.

A major issue for healing chronic wounds is that the hypoxic conditions caused by vascular complications inhibit healing.^3^ Molecular oxygen is essential during the inflammatory phase of wound healing and during collagen synthesis.^4^ Wound treatment with oxygen increases vascular endothelial growth factor (VEGF) expression at wound edges^5^ and increases angiogenesis.^6^ Chronic wounds have lower tissue oxygen tensions than control tissues, a predictor of extremity chronic wound healing.^7,8^

One approach to improving outcomes for chronic wound patients is treatment with oxygen therapy. One example is hyperbaric oxygen therapy (HBOT), which requires the patient to enter a chamber pressurized to 2–2.5 atm with 100% oxygen for 90–120 min daily for 2–8 weeks. HBOT appears to be effective as an adjunctive therapy for many patients.^9,10^ However, there are drawbacks to HBOT, including oxygen toxicity, barotrauma, pneumothorax, risk of fire and explosion, and inconvenience of accessing a clinic equipped with a chamber.

Topical oxygen therapy (TOT) has none of the associated risks and drawbacks of HBOT, and has been shown to be a safe and effective treatment option for many chronic wound patients.^5,11–13^ TOT is Food and Drug Administration (FDA) cleared for skin ulcerations due to diabetes, venous stasis, postsurgical infections and gangrenous lesions, decubitus ulcers; amputations/infected stumps; skin grafts; burns; and frostbite. The disposable, single-use devices are self-administered by the patient permitting at-home use of TOT.

Several clinical studies have demonstrated TOT effectiveness. A retrospective study on 58 wounds in 32 patients treated with TOT had a healing rate of 65.5%.^12^ These results have been validated in small cohorts of chronic wound patients, where the wounds experienced some healing during treatment.^14–16^

In one study, patients with venous leg ulcers were treated with TOT (*n* = 46) or conventional compression dressings (*n* = 37). The study found 80% of wounds treated with TOT healed compared to 35% of wounds treated with conventional compression dressings.^13^ Another study compared HBOT (32 patients) to TOT (25 patients) on chronic wound healing after 12 weeks of therapy. TOT decreased wound volume, while no improvement was observed in patients who received HBOT.^5^ Wound edge tissue biopsies treated with TOT had increased expression of VEGF,^5^ an important growth factor supporting angiogenesis. In another study, TOT (67 patients) was compared to conventional compression dressing (65 patients) for 12 weeks or until full healing. Wounds treated with TOT experienced a 96% reduction in wound area, while wounds treated with conventional compression dressing experienced a 61% reduction in wound area.^11^ At 12 weeks, 67% of patients treated with TOT healed completely, while 46% of patients treated with conventional compression dressing healed fully.^11^

## Clinical Problem Addressed

The current data for the effectiveness of TOT on chronic wound healing come from controlled studies performed in clinical settings. The purpose of this retrospective chart review was to determine if TOT has a similar level of effectiveness in healing chronic wounds during routine clinical use in the at-home treatment setting. This study was designed to investigate the factors that influence chronic wound healing and amputation rates in wounds treated with TOT. The analysis considered variables, including patient age, patient gender, primary payer type, diagnosis/International Classification of Diseases (ICD)9 code, wound(s) location, wound(s) age, treatment duration, dimensions of the wound(s) before and after treatment with TOT, and the reason for ending TOT.

## Materials and Methods

### Internal Review Board

This retrospective, descriptive chart review was approved by the Internal Review Board (IRB) Company (Buena Park, CA) under exempt status (IRB No.: 2016-0022-GWR).

### Participants

Charts from male and female subjects ranging in age from 4 years to 105 years were reviewed as part of this study. This retrospective chart review was performed under a waiver of consent due to the minimal risk to patients and the absence of patient identifiers in the data set.

### GWR Medical database

The patient records reviewed for this study were extracted from a database owned and maintained by GWR Medical, Inc. Physicians who prescribed GWR TOT submitted the data contained in this database. The data are submitted for approval for GWR Medical to release the TOT to the patient and for billing purposes. This database contained records corresponding to a total of 10,980 wounds at the time the data were reviewed for inclusion in this study. All of the records were reviewed against the inclusion criteria for this study. A total of 3,462 patient records, representing 4,127 total wounds, were included in this study. As a measure to ensure patient privacy, the principal investigator (PI) and statistician assigned to this study had no access to this database or any patient health information associated with the records. The relevant data used in this study were extracted from the GWR database, fully deidentified by a member of the GWR Medical team, and sent to the PI and statistician in a locked Excel spreadsheet. The data elements used in this study were restricted to patient age at the time of treatment, patient sex, primary insurance, primary and/or secondary diagnosis and ICD9 code, wound(s) location(s), wound(s) age(s), treatment duration, dimensions of the wound(s) before and after treatment with topical oxygen, and the reason for ending topical oxygen treatment.

### Inclusion criteria

The wound records included in this retrospective chart review were collected between January 1, 2007, and July 18, 2016. All of the wound records included in this study were collected and completed before the initiation of this study. All included records were from wounds that were no longer being treated with TOT. Records that were missing any of the data elements collected as part of this study were excluded from this study. Records for wounds from male and female patients in the age range of 4 years to 105 years were included in this study. All patients had at least one chronic wound.

All patients were treated with TOT in compliance with the FDA-approved indications for use of this therapy. TOT is approved for treatment of open acute or chronic wounds, for example, skin ulcerations due to diabetes, venous stasis, postsurgical infections and gangrenous lesions; decubitus ulcers; amputations/infected stumps; skin grafts; burns; and frostbite. All patients included were treated with at least one separate modality before treatment with TOT and had completed the TOT treatment to be included in this study. All of the wounds were at least 1 cm^2^ and treated with TOT for a minimum of 2 weeks to be included in this study.

### Wound assessment details

Clinical assessment of the wounds was performed by trained medical personnel. Wound assessments were sent to GWR Medical for inclusion in their database. Wound measurements (length and width) in centimeters were collected for each wound before initiation of TOT and again after termination of TOT. The measurements were either collected by the medical personnel assessing the wounds or were taken from photographs of the wounds and sent to GWR Medical. In the cases where photographs were used, wound measurements were calculated using WoundMatrix, an FDA-listed 21 Code of Federal Regulations (CFR) Part 11 compliant software package, which uses digital planimetry to calculate wound measurements (WoundMatrix, Inc. Chadds Ford, PA).

### Wound healing rate calculation

Wound healing after TOT was assessed using the following equation:

Wound healing = (Wound area before TOT − Wound area after TOT)/Wound area before TOT. Wound healing was expressed as a percentage for each wound.

### TOT regimen

TOT was administered to all patients who met the FDA-approved indications for use of TOT [510(k) No. K971507]. The area of the patient's wound was fitted with GWR Medical's single-use, disposable O_2_Boot or O_2_Sacral device. The area surrounding the wound received 100% oxygen at 1.03 atm for 90 min for 4 consecutive days, followed by 3 days without treatment.^17^ The weekly treatment regimen was self-administered in the patient's home and continued as directed by the healthcare provider.

### Data analysis

The data analysis focus was on descriptive statistics to describe the healing and amputation rates of the wounds. All analyses were on a per wound basis, rather than by individual patient. More than 85% of patients in the included data set only had a record for one wound. *p*-Values from Pearson's chi-squared tests for response homogeneity are reported (Table 2) to provide a measure of statistical significance for the difference in response distributions across groups. We note that the *p*-values should be interpreted with caution as the large database allows for the identification of small effects as statistically significant. Other specific tests used are noted in the results.

The decision to focus on categorical data analysis was made due to the structure of the data. The data were obtained retrospectively from a historical database. Using categories, as opposed to continuous data values, guards against data quality issues (*e.g.* transcription errors) that are not unusual in such databases.

Categorical groups for patient age, wound size, wound age, and amount of healing were used in the analysis. The groups were based on data collected on numerical scales. Age groups were defined as children and adolescents, <21, adults 21–64, and older adults 65+. Wound size categories were defined by the quartiles of the recorded starting wound size as area in cm^2^. Wound age was defined as 1, 2–3, 4–12, and >12 months. The primary insurance type was grouped into Medicare, Medicaid, Private, and other. The amount of healing was defined as 100% healed (as identified by a physician or by measurement), some healing (ending wound area was smaller than the starting wound area), and no healing (ending wound size was equal to or greater than the starting wound area). Analysis was performed using JMP Pro 13 (SAS Institute, Cary, NC).

## Results

### Patient demographics and clinical variables

The 4,127 chronic wounds included in this study were from patients who ranged in age at the time of treatment from 4 years to 105 years with a median age of 60. Table 1 provides a summary of wounds based on gender, age groups, primary insurance, and primary diagnosis (ICD9 code). Wound records from male (55.9%) and female (44.1%) patients were included in this study. The majority of wounds were associated with one of four diagnoses: diabetes mellitus (ICD9 250, 34.2%), other disorders of the circulatory system (ICD9 459, 11%), chronic ulcer of the skin (ICD9 707, 27.2%), and/or complication of surgical and medical care (ICD9 998, 6%). More than 80% of wounds associated with an initial ICD9 code of 250 or 459 had ICD9 707 (chronic ulcer of the skin) as a secondary diagnosis. A total of 42.3% of the wounds included in this chart review were from Medicare (Primary insurance) eligible patients, while 35.6% of these wounds were from Medicaid (Primary insurance) eligible patients.

**Table 1.** Patient demographics and clinical variables by wound

	N	*%*
Total wounds	4,127	100
Gender		
Male	2,305	55.9
Female	1,822	44.1
Age group		
<21	20	0.5
21–64	1,967	47.7
65+	2,140	51.8
Insurance		
Medicare	1,747	42.3
Medicaid	1,467	35.6
Private	556	13.5
Other	357	8.7
Diagnosis 1 ICD9 code		
ICD9 250: Diabetes mellitus	1,410	34.2
ICD9 459: Other disorders of circulatory system	454	11.0
ICD9 707: Chronic ulcer of skin	1,123	27.2
ICD9 998: Complication of surgical and medical care, not otherwise specified	246	6.0
Other	894	21.7
Healing		
No healing	1,674	40.6
Some healing	1,316	31.9
100% Healed	1,137	27.5
Reason for ending treatment		
Wound healed	1,619	39.2
Doctor/patient discontinued	807	19.6
Undetermined/other	749	18.1
Patient hospitalized	370	9.0
Coverage denied	220	5.3
Noncompliant patient	169	4.1
Hospitalized—amputation	97	2.3
Patient passed away	96	2.3

ICD, International Classification of Diseases.

### Wound characteristics

Chronic wounds ranged in age from 1 month to over 1 year (Table 2). The foot was the most common wound location (46%) followed by the leg (20.7%), while the sacral region (2.2%) was the least represented site. Most of the wounds on the foot (50.5%) and toe (44.0%) were associated with patients having a diabetes mellitus diagnosis (ICD9 250). The majority of the sacral wounds (55.7%) were associated with patients having a diagnosis of chronic ulcer of the skin.

**Table 2.** Wound characteristics

	N	*%*
Total wounds	4,127	100
Wound age		
1 Month	1,028	24.9
2–3 Months	1,057	25.6
4–12 Months	1,475	35.7
>12 Months	567	13.7
Wound starting size		
<2.5 cm^2^	1,039	25.2
2.5–6.0 cm^2^	1,040	25.2
6.1–16 cm^2^	1,042	25.2
>16 cm^2^	1,006	24.4
Wound ending size^a^		
<2.5 cm^2^	2,210	53.5
2.5–6.0 cm^2^	651	15.8
6.1–16 cm^2^	593	14.4
>16 cm^2^	669	16.2
Wound location^b^		
Foot	1,900	46.0
Leg	855	20.7
Other or not specified	504	12.2
Ankle	393	9.5
Toe	386	9.4
Sacral	89	2.2

^a^ Data for four wounds were missing ending measurements.

^b^ Wound location was derived from location description and/or diagnosis notes.

Initial wound size ranged in total area from 1 to 1,500 cm^2^ with a median area of 6 cm^2^. A total of 24.4% of the wounds had a wound starting size >16 cm^2^. Following TOT, this group of wounds (>16 cm^2^) contained only 16.2% of the wounds (Table 2), suggesting that many of the largest, chronic wounds experienced a healing benefit after TOT. The change in total wound area after TOT ranged from −2,717% to 100%. The large negative changes tended to be for wounds with a smaller initial size that did not heal (5% of wounds in this study). The majority of the wounds treated with TOT had a decrease in size after treatment (59.4% of all wounds in the study). At the end of treatment, the median wound size was 2 cm^2^ with 27.5% of wounds being completely healed (identified by a physician or measurement).

### Reason for ending TOT

TOT is convenient and easy to use. Patients can self-administer TOT at home rather than traveling to a healthcare facility. In this study, there was a noncompliance rate of 4.1% (169 wounds), which may reflect the convenience of at-home, self-administration of TOT.

**Figure. 1.**
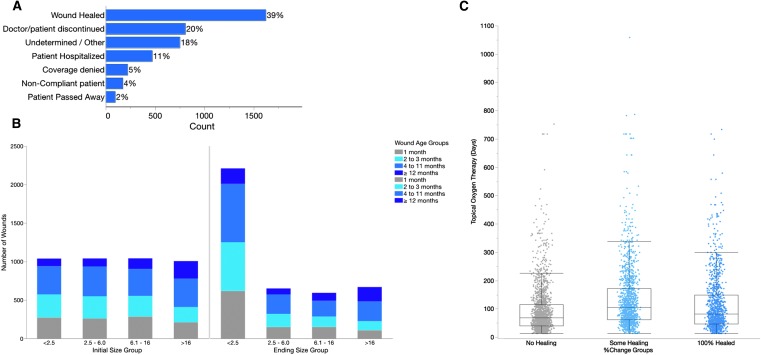
Chronic wound treatment with TOT. **(A)** Reasons for ending TOT plotted by frequency, **(B)** wound size before and after treatment with TOT, **(C)** duration of TOT as a function of initial wound size grouping. TOT, topical oxygen therapy.

In a total of 5.3% of cases where TOT was terminated, the reason given was coverage denial by insurance for TOT (Fig. 1A). Wound healing was the most frequent reason for ending TOT with a total of 1,619 wounds terminating TOT because of healing (39.2%). When wound size before and after administration of TOT was considered, the number of wounds <2.5 cm^2^ increased from 1,039 (25.2%) to 2,210 (53.5%) (Fig. 1B), evidence of wound healing following TOT.

### Wound healing

Overall, treatment of chronic wounds with TOT resulted in at least some healing for 59.4% of the wounds included in this study (Table 1). Wounds that did not heal were treated for the shortest time (median 69 days) when compared to wounds that experienced some healing (median 105 days) and 100% healing (median 82 days) (Fig. 1C). These differences are statistically significant (Steel–Dwass method *p*-values <0.0001 for all pairs).

Tables 3 and 4 are a compilation of cross tabulations. Each section of the table includes the counts, column percentages, and row percentages to compare the levels of the grouping variable to the amount of healing. We considered that variables related to the chronic wound itself and variable related to the patient may impact wound healing following treatment with TOT.

**Table 3.** Wound characteristics impacting wound healing following tot, 2 × 2 cross tabulations with tests for response homogeneity

	*No Healing*	*Some Healing*	*100% Healed*		
	*Count; Col%; Row%*			*Total*	p^a^
Wound age groups					
1 Month	351; 20.97%; 34.14%	319; 24.24%; 31.03%	358; 31.49%; 34.82%	1,028	<0.0001
2–3 Months	408; 24.37%; 38.60%	324; 24.62%; 30.65%	325; 28.58%; 30.75%	1,057	
4–12 Months	604; 36.08%; 40.95%	522; 39.67%; 35.39%	349; 30.69%; 23.66%	1,475	
>12 Months	311; 18.58%; 54.85%	151; 11.47%; 26.63%	105; 9.23%; 18.52%	567	
Total	1,674	1,316	1,137	4,127	
Initial size group					
<2.5	391; 23.36%; 37.63%	277; 21.05%; 26.66%	371; 32.63%; 35.71%	1,039	<0.0001
2.5–6.0	420; 25.09%; 40.38%	335; 25.46%; 32.21%	285; 25.07%; 27.40%	1,040	
6.1–16	385; 23.00%; 36.95%	365; 27.74%; 35.03%	292; 25.68%; 28.02%	1,042	
>16	478; 28.55%; 47.51%	339; 25.76%; 33.70%	189; 16.62%; 18.79%	1,006	
Total	1,674	1,316	1,137	4,127	
Wound site					
Ankle	172; 10.27%; 43.77%	121; 9.19%; 30.79%	100; 8.80%; 25.45%	393	<0.0001
Foot	696; 41.58%; 36.63%	665; 50.53%; 35.00%	539; 47.41%; 28.37%	1,900	
Leg	381; 22.76%; 44.56%	263; 19.98%; 30.76%	211; 18.56%; 24.68%	855	
Other	220; 13.14%; 43.65%	148; 11.25%; 29.37%	136; 11.96%; 26.98%	504	
Sacral	51; 3.05%; 57.30%	15; 1.14%; 16.85%	23; 2.02%; 25.84%	89	
Toe	154; 9.20%; 39.90%	104; 7.90%; 26.94%	128; 11.26%; 33.16%	386	
Total	1,674	1,316	1,137	4,127	

^a^ *p*-Value from Pearson's chi-squared test of response homogeneity.

**Table 4.** Patient characteristics impacting wound healing following tot, 2 × 2 cross tabulations with tests for response homogeneity

	*No Healing*	*Some Healing*	*100% Healed*		
	*Count; Col%; Row%*			*Total*	p^a^
ICD9 group					
250	465; 27.78%; 32.98%	579; 44.00%; 41.06%	366; 32.19%; 25.96%	1,410	<0.0001
459	159; 9.50%; 35.02%	198; 15.05%; 43.61%	97; 8.53%; 21.37%	454	
707	560; 33.45%; 49.87%	206; 15.65%; 18.34%	357; 31.40%; 31.79%	1,123	
998	91; 5.44%; 36.99%	73; 5.55%; 29.67%	82; 7.21%; 33.33%	246	
Other	399; 23.84%; 44.63%	260; 19.76%; 29.08%	235; 20.67%; 26.29%	894	
Total	1,674	1,316	1,137	4,127	
Age group					
<21	4; 0.24%; 20.00%	8; 0.61%; 40.00%	8; 0.70%; 40.00%	20	<0.0001
21–64	690; 41.22%; 35.08%	750; 56.99%; 38.13%	527; 46.35%; 26.79%	1,967	
65+	980; 58.54%; 45.79%	558; 42.40%; 26.07%	602; 52.95%; 28.13%	2,140	
Total	1,674	1,316	1,137	4,127	
Insurance group					
Medicaid	409; 24.43%; 27.88%	698; 53.04%; 47.58%	360; 31.66%; 24.54%	1,467	<0.0001
Medicare	786; 46.95%; 44.99%	487; 37.01%; 27.88%	474; 41.69%; 27.13%	1,747	
Other	192; 11.47%; 53.78%	39; 2.96%; 10.92%	126; 11.08%; 35.29%	357	
Private	287; 17.14%; 51.62%	92; 6.99%; 16.55%	177; 15.57%; 31.83%	556	
Total	1,674	1,316	1,137	4,127	

^a^ *p*-Value from Pearson's chi-squared test of response homogeneity.

Wound healing was impacted by wound-dependent variables, including wound age, initial wound size, and wound site. Wounds less than 1 year old were the most likely to experience healing when treated with TOT (Table 3) with >50% of all wounds less than 1 year old experiencing healing after TOT (Table 3, Row% Some Healing +100% Healed). Older wounds (>12 months) had a no healing rate of 54.9%, the highest rate of no healing for all of the wound age groups (Table 3, Row%). The analysis was performed between the groups to determine the impact of wound age on wound healing (Table 3, Col%). By this analysis, the wounds that were ≤12 months old were more likely to heal than wounds >12 months old (Table 3, Col% Some Healing +100% Healed, *p* < 0.0001).

Wound healing during treatment with TOT was associated with wound size before initiating TOT. More than 50% of chronic wounds, regardless of their size before starting TOT, had some healing following TOT (Table 3, Row% Some Healing +100% Healed). The chronic wounds that were less than 16 cm^2^ experienced the greatest rate of healing after TOT (Table 3, Row% Some Healing +100% Healed), with the smallest wounds (<2.5 cm^2^) experiencing the highest rate of complete healing (Table 3, Row%). The largest wounds (>16 cm^2^) were the least likely to heal with only an 18.8% complete healing rate (Table 3, Row%). These differences are statistically significant (Steel–Dwass method *p*-values <0.0001 for all pairs). The impact of initial wound size on wound healing was analyzed between the groups (Table 3, Col%). Wounds that were <2.5 cm^2^ were more likely to experience 100% healing than wounds in any other group, while the largest wounds (>16 cm^2^) were the least likely to experience 100% healing (Table 3, Col%, *p* < 0.0001).

Chronic wounds at all locations had some healing for >50% of the wounds in this study with the exception of sacral wounds, which tended to be larger before initiating the therapy (Table 3, Row%). Wounds located on the foot and the toe had higher healing rates after TOT (63.4% and 60.1%, respectively) when compared to other sites (Table 3, Row% Some Healing +100% Healed; *p*-values <0.0001 for all pairs). The impact of wound location on wound healing was analyzed between the groups (Table 3, Col%). Wounds located on the foot were more likely to experience 100% healing (47.41%) than wounds at other locations on the body, while sacral wounds were the least likely to experience 100% healing (Table 3, Col%, *p* < 0.0001).

Wound healing was impacted by patient-dependent variables, including patient diagnosis, patient age, and insurance type. Wounds from patients with a diagnosis of diabetes mellitus (ICD9 250) were the most likely to experience partial or complete healing (67.1%, *n* = 945) following TOT (Table 4, Row% Some Healing +100% Healing) when compared to other diagnoses (*p* ≤ 0.0001). Wounds from patients with a diagnosis of chronic ulcer of the skin (ICD9 707) had the lowest rate of wound healing with 50.1% after TOT (Table 4, Row%; *p* ≤ 0.0001). Wounds from patients with a diagnosis of chronic ulcer of the skin (ICD9 707) that experienced healing, tended to be from slightly older than patients whose wounds experienced some healing (median age 64 for no healing group, median age 60 for some healing group, and median age 61 for 100% healing group).

Chronic wounds from patients of all age groups experienced a benefit from TOT with >50% of all wounds experiencing at least some healing (Table 4, Row% Some Healing +100% Healing; *p* ≤ 0.0001). The majority of wounds that experienced 100% healing were associated with patients who were older than 65 (Table 4, Col%). Wounds from patients eligible for Medicare had a healing rate of 55% after TOT (Table 4, Row%, Some Healing +100% Healing). A similar rate of wound healing (72.1%) was observed for wounds from patients receiving Medicaid services (Table 4, Row%, Some Healing +100% Healing). These healing rates were statistically significant (*p* < 0.0001).

### Amputation rate

The amputation rate for wounds treated with TOT was 2.4% (Table 5). Out of the 4,127 wounds treated with TOT, only 97 of those wounds were associated with amputation. The amputation rate was independent of patient gender and the wound starting size (Table 5, *p* = 0.6348).

**Table 5.** Amputation rate following treatment of chronic wounds with tot with tests for response homogeneity

	*N (Amputations)/N (Group)*	*Amputation Rate (%)*	p^a^
Overall	97/4,127	2.4	NA
Gender			
Male	47/2,305	2.0	0.1479
Female	50/1,822	2.7	
Wound age			
1 Month	27/1,028	2.6	<0.0001
2–3 Months	43/1,057	4.1	
4–12 Months	19/1,475	1.3	
>12 Months	8/567	1.4	
Wound starting size			
<2.5 cm^2^	25/1,039	2.4	0.6348
2.5–6.0 cm^2^	20/1,040	1.9	
6.1–16 cm^2^	29/1,042	2.8	
>16 cm^2^	23/1,006	2.3	
Insurance			
Medicaid	19/1,467	1.3	0.0002
Medicare	49/1,747	2.8	
Other	5/357	1.4	
Private	24/556	4.3	
Wound location^a^			
Foot	50/1,900	2.6	0.0002
Leg	12/855	1.4	
Other or not specified	12/504	2.4	
Ankle	2/393	0.5	
Toe	20/386	5.2	
Sacral	1/89	1.1	
ICD9 code			
ICD9 250: Diabetes mellitus	45/1,410	3.2	0.0137
ICD9 459: Other disorders of circulatory system	2/454	0.4	
ICD9 707: Chronic ulcer of skin	28/1,123	2.5	
ICD9 998: Complication of surgical and medical care, not otherwise specified	5/246	2.0	
Other	17/894	1.9	

^a^ *p*-Value from Pearson's chi-squared test of response homogeneity.

The amputation rates differed statistically by wound location (Table 5, *p* = 0.0002), with the highest rate of amputation being for wounds located on the toe (5.2%). Amputation rates differed statistically by ICD9 code (Table 5, *p* = 0.0137). Chronic wounds associated with patients who had a diagnosis of other disorders of the circulatory system (ICD9 459) had a very low amputation rate of 0.4% (Table 5). A higher rate of wounds (3.2%) associated with patients with a diabetes mellitus diagnosis (ICD9 250) resulted in an amputation (Table 5).

Chronic wounds associated with patients covered by private insurance had the highest rate of amputation (4.3%), while chronic wounds associated with patients covered by Medicare or Medicaid had amputation rates close to the amputation rate observed for all chronic wounds in this study (Table 5, *p* = 0.0002).

## Discussion

The chronic wounds included in this retrospective chart review had similar rates of healing (59.4%) and amputation (2.4%) after treatment with TOT to those observed in other studies.^5,12^ The healing rate included both 100% healed wounds and partially healed wounds. Wound healing was reflected in the most frequent reason for ending TOT (39.2%) and in the number of wounds with a reduction in size or a statement from the physician that the wound healed (59.4%). Chronic wounds that were smaller and less than 1 year old experienced the greatest healing benefit from TOT (Table 3). Although the data set was smaller for sacral wounds (2.2% of the total chronic wounds in this study), they tended to be larger and experienced less healing than wounds at other locations. Overall, 26% of sacral wounds experienced 100% healing. Recent case studies have reported wound healing in patients with sacral pressure wounds associated with^18^ and without spinal cord injury.^19^ A more thorough investigation, including a larger sample size of chronic sacral wounds, is needed to understand the factors impacting the effectiveness of TOT for sacral wounds. However, the data from this study and previous studies suggest that topical oxygen may be a promising treatment modality for these wounds.^18,19^

When the treatment time was considered for healing and nonhealing wounds, we discovered that wounds that did not heal were treated with TOT for the shortest time (median 69 days) when compared to wounds that experienced some healing (median 105 days) and 100% healing (median 82 days). This difference is statistically significant (*p*-values <0.001 for Steel–Dwass multiple comparison test). Perhaps some of the wounds that did not heal would have had a better outcome if the TOT regimen had been extended. This is an interesting area for future investigation.

These data demonstrate that TOT can support complete wound closure and help reduce the size of the wound to allow resumption of standard wound care. As a retrospective chart review, this study does have some limitations. First, the data set did not include information on wound recurrence after cessation of TOT. Previous controlled studies have shown a low rate of wound recurrence following treatment of chronic wounds with TOT.^13,16^ As a retrospective chart review of wounds treated only with TOT, this study does not compare effectiveness of TOT to other treatment modalities (*e.g.*, HBOT) in a controlled setting. However, numerous controlled clinical studies comparing TOT to other treatment modalities have been performed previously.^5,11,13^

Although this study did not have access to data regarding pain reduction as these wounds healed, a recent study measured pain as an endpoint in a cohort of patients with refractory venous ulcers treated with TOT. After 13 days of treatment, the patients receiving TOT reported a decrease from 8 to 3 on the pain numerical ranking scale.^11^ The data presented here and in similar studies indicate that TOT has a positive impact on chronic wound healing, reduces the rate of amputation, and leads to better quality of life for patients.

## Innovation

The results of this large retrospective chart review validate the healing rates and amputation rates observed in controlled clinical studies using TOT to manage chronic wounds of various etiologies. This study demonstrates improved outcomes measured by a high healing rate (59.4% for 100% healing and some healing) and low amputation rates (2.4%).

Key Findings

• Chronic wounds treated with TOT had a rate of healing similar to healing rates observed in controlled clinical trials.
• Chronic wounds treated with TOT had a low rate of amputation, regardless of site, wound age, wound starting size, and other clinical variables.
• The results from this study demonstrate improved patient outcomes and improved quality of life, similar to results from other studies, when chronic wounds are treated with TOT.

## About the Authors

**Angie R. Purvis, PhD,** received her BS in Biology from Centre College and her PhD in biochemistry from Washington University. Her PhD thesis and her postdoctoral training at Washington University were in the field of hematology/hemostasis. She works as an independent scientific consultant across the biotechnology industry. **Karen Copeland, PhD,** received her BA from St. Olaf College. She received her MS and PhD in mathematical sciences from Clemson University. She works as an independent statistical consultant across the biotechnology industry.

## Abbreviations and Acronyms

CFR: Code of Federal Regulations
FDA: Food and Drug Administration
HBOT: hyperbaric oxygen therapy
IRB: Internal Review Board
ICD: International Classification of Diseases
PI: principal investigator
TOT: topical oxygen therapy
VEGF: vascular endothelial growth factor
